# Letter to the Editor: Comments on “Obesity associated with a novel mitochondrial tRNA^Cys^ m.5802A>G mutation in a Chinese family”

**DOI:** 10.1042/BSR20194502

**Published:** 2020-02-11

**Authors:** Josef Finsterer

**Affiliations:** Krankenanstalt Rudolfstiftung, Messerli Institute, Austria

**Keywords:** mitochondrial, mtdna, mutation, obesity, tRNA

## Abstract

In a recent article Wang et al. reported about a 9-year-old Chinese male with obesity, insulin resistance, acanthosis nigricans, hyperuricemia, and non-alcoholic fatty liver disease (NALFD) being attributed to the variant m.5802A>G in *tRNA(Cys)* (*Bioscience Reports* (2020) **40**(1), BSR20192153). Pathogenicity of the variant was substantiated by documentation of perturbed stability and mobility of the tRNA(Cys). The interesting study has a number of shortcomings, which do not allow drawing conclusions as provided before they are solved. Obesity is multifactorial and many differential causes of mtDNA variants were not discussed or excluded.

Dear Editor,

With interest we read the article by Wang et al. about a 9-year-old Chinese male with obesity, insulin resistance, acanthosis nigricans, hyperuricemia, and non-alcoholic fatty liver disease (NALFD) being attributed to the variant m.5802A>G in *tRNA(Cys)* [1]. Pathogenicity of the variant was substantiated by documentation of perturbed stability and mobility of the tRNA(Cys) [1]. The study has a number of shortcomings.

The first shortcoming of the study is that the heteroplasmy rate of the m.5208A>G variant was not provided. Knowing heteroplasmy rates in different tissues is crucial for assessing the pathogenicity, for genetic counselling of family members, and for assessing the prognosis of the disease. We should know heteroplasmy rates in hair follicles, buccal mucosa cells, fibroblasts, muscle, lymphocytes, and urinary epithelial cells form the index patient and all mutation carriers.

The second shortcoming of the study is that the pathogenicity of the variant was not convincingly confirmed. To confirm that the variant was truly responsible for familial obesity in this pedigree, the modified Yarham score should be applied [2]. According to the modified Yarham score, the variant m.5208A>G scored only 2 points, implying that the variant is only possibly pathogenic (>1 independent report: 0; heteroplasmy: 0; disease segregation with variant: 0; variant segregation with biochemical defect in single-fiber studies: 0; mutant mt-tRNA steady-state level studies or evidence of pathogenicity in *trans*-mitochondrial cybrid studies: 0; evidence of normality in *trans*-mitochondrial cybrid studies: 0; evolutionary conservation of nucleotide: 2; mitochondrial histopathology: 0) [2].

A further argument against the pathogenicity of the variant m.5802A>G is that the described patient manifested only with obesity, hyperuricemia, steatosis, insulin resistance, and acanthosis nigricans. However, other typical clinical manifestations of a mitochondrial disorder (MID) were not reported. Since MIDs are usually multisystem diseases affecting not only a single organ or tissue [3], the clinical presentation of the index case argues against pathogenicity. Acanthosis nigricans has not been reported in association with a MID.

Missing in this report is serum and cerebrospinal fluid (CSF) lactate values. MIDs frequently manifest with lactic acidosis, which determines the course and outcome. Thus, we should know if serum/CSF lactate values were elevated in the index patient or other mutation carriers. Additionally, we should be informed about results of the lactate stress test (LST) [4], which has been shown to be a reliable screening test for the presence/absence of an MID.

A further shortcoming is that the nutrition habits of the index patient and his family were not provided. Missing in this respect is also the exclusion of causes other than the mtDNA variant for obesity, such as hormone levels, and function of pituitary, thyroid, and suprarenal glands.

We do not agree with the notion that “tRNA mutations are particularly associated with enhanced childhood obesity risk” [1]. On the contrary, mtDNA mutations are more frequently associated with weight loss than with weight gain.

Furthermore, conformational changes or impaired stability or mobility of the tRNA molecule not necessarily imply perturbed function of the tRNA [5].

Overall, this interesting case report has a number of shortcomings, which do not allow drawing conclusions as provided before they are solved. Obesity is multifactorial and many differential causes of mtDNA variants were not discussed or excluded.

## Abbreviations

CSF: cerebrospinal fluid
LST: lactate stress test
MID: mitochondrial disorder
NALFD: non-alcoholic fatty liver disease
